# Clinical Characteristics of Arginase 1 Deficiency: Natural History Insights From International Clinical Trials

**DOI:** 10.1002/jimd.70156

**Published:** 2026-02-06

**Authors:** Mattias Rudebeck, Nancy Braverman, Richard Chang, Gregory M. Enns, Arunabha Ghosh, Magali Gorce, Daniela Karall, Reena Sharma, Emily Shelkowitz, Roberto Zori, Markey McNutt

**Affiliations:** ^1^ Immedica Pharma AB Stockholm Sweden; ^2^ Research Institute of the McGill University Health Center Montreal Quebec Canada; ^3^ Rady Children's Health, Orange County Orange California USA; ^4^ Stanford University School of Medicine and Lucille Packard Children's Hospital Stanford California USA; ^5^ Willink Biochemical Genetics Unit Manchester University Foundation Trust Manchester UK; ^6^ School of Biological Sciences University of Manchester Manchester UK; ^7^ Hôpital Des Enfants, CHU Toulouse Toulouse France; ^8^ Klinik Für Pädiatrie Medizinische Universität Innsbruck Innsbruck Austria; ^9^ Department of Adult Inherited Metabolic Disorders Salford Royal NHS Salford UK; ^10^ University of Washington Seattle Washington, DC USA; ^11^ University of Florida Gainesville Florida USA; ^12^ UT Southwestern Medical Center Dallas Texas USA

**Keywords:** ARG1‐D, arginase 1, disease characteristics, natural history, urea cycle disorders

## Abstract

Arginase 1 deficiency (ARG1‐D) is an ultra‐rare inherited metabolic disorder of the urea cycle, caused by partial or complete loss of arginase 1 function, characterised by hyperargininaemia and a distinct, progressive neurological phenotype. The clinical development programme of pegzilarginase, a recombinant human ARG1 enzyme therapy, provides an opportunity to study the largest ARG1‐D cohort to date. The analysis included 48 paediatric and adult subjects (≥ 2 years) enrolled in the pegzilarginase trials. Baseline data collected before treatment included demographics, genotypes, red blood cell arginase activity, biochemical measures, age of symptom onset, neuromotor and neurological characteristics, growth indicators, quality of life, and use of treatments and assistive devices. The mean (SD) age of onset was 2.2 (3.6) years, which preceded diagnosis at 3.7 (5.0) years. Clinical features included motor impairment (48/48, 100%), spasticity (33/48, 69%), cognitive deficits (31/48, 65%), intellectual disability (23/36, 64%), speech and language deficits (26/48, 54%), and seizures (18/48, 38%), with symptom‐onset data consistent with a progressive phenotype. Median GMFCS Level II indicated moderate mobility limitation; two‐thirds scored < 69 on FSIQ, and mean PedsQL total proxy scores were around 20% lower than typically developing peers. All subjects followed a protein‐restricted diet, and 90% used ammonia scavengers. ARG1‐D presents with a heterogeneous array of progressive and debilitating neurologic symptoms. These findings reflect the progressive impact of the disease and offer insights into its burden and natural history based on a large cohort, assessed using standardised neuromotor, cognitive, and quality‐of‐life instruments across international sites.

## 1 Introduction

Arginase 1 deficiency (ARG1‐D) is a rare inherited metabolic disorder caused by autosomal recessive pathogenic variants of the *ARG1* gene, resulting in partial or complete loss of arginase 1 function [1, 2, 3]. This impairs the terminal step of the hepatic urea cycle, preventing the conversion of arginine to ornithine and urea, and causing accumulation of plasma arginine (pArg) and its metabolites: the guanidino compounds (GCs). Distinct from other urea cycle disorders (UCDs), hyperargininaemia is the characteristic biochemical and pathological abnormality, typically manifesting around 2–4 years of age as a distinct and progressively debilitating neurological phenotype [4].

Progressive lower‐limb spasticity is the hallmark feature of ARG1‐D, leading to frequent misdiagnosis as other neurological movement disorders such as cerebral palsy (CP) and hereditary spastic paraplegia [5, 6, 7]. Persistently elevated plasma arginine can help distinguish ARG1‐D from these conditions. Patients typically present with gait abnormalities, difficulty walking and climbing stairs, and often require assistive devices. Other symptoms appearing within the first few years of life include delayed development, dietary protein intolerance, and vomiting [8, 9, 10, 11].

Without treatment, prolonged exposure to excessive arginine and GCs exacerbates neurological manifestations [12, 13, 14, 15]. Most patients develop gross motor dysfunction and impaired mobility, often becoming non‐ambulatory, and may experience severe cognitive impairment, intellectual disability, and seizures.

The estimated birth prevalence of ARG1‐D ranges from approximately 1 in 198 000 to 1 in 950 000, depending on demographics and consanguinity [16, 17, 18, 19]. The burden of disease is severe, with patients requiring continuous treatment and support throughout life [18]. Current standard of care involves a low‐protein arginine‐restricted diet, supplemented with essential amino acids, to maintain plasma arginine as close to normal as possible, or failing that, ≤ 200 μmol/L [13, 20, 21]. Nitrogen scavengers such as glycerol phenylbutyrate, sodium phenylbutyrate, and sodium benzoate may be used to facilitate alternative nitrogen excretion and reduce hyperammonaemic risk. The long‐term prognosis is generally poor; ARG1‐D has been reported to be associated with significant morbidity and early mortality (< 40 years) [22]. Accordingly, an effective disease‐modifying treatment which slows disease progression and improves clinical outcomes has long been needed [13].

Pegzilarginase (Loargys, Immedica Pharma AB) is a pegylated and cobalt‐substituted recombinant human ARG1 enzyme therapy [23, 24] shown in clinical trials to normalise plasma arginine and improve clinical outcomes [24, 25]. Baseline assessments from these trials, conducted using standardised neuromotor, cognitive, and quality‐of‐life measures, provide insights into the natural history and clinical characteristics of ARG1‐D.

## 2 Methods

### 2.1 Patient Populations and Study Designs

Patients were part of the clinical development program for pegzilarginase, which comprised three studies. The Phase 1/2 open‐label dose escalation study (Study 101A) which was followed by a long‐term extension study in the same subjects [23] and the Phase 3 Pegzilarginase Effect on Arginase 1 Clinical Endpoints (PEACE) trial (Study 300A), which was a randomised, double‐blind placebo‐controlled study [24, 25]. A total of 48 patients were enrolled across the Phase 1/2 study and PEACE [23, 24]. In the Phase 1/2 study, 16 patients were recruited across nine sites in four countries (Canada, Portugal, UK and US). The PEACE study recruited 32 patients and included 19 sites in seven countries (Austria, Canada, France, Germany, Italy, UK, and US). Both studies included adult and paediatric patients ≥ 2 years of age, with a confirmed diagnosis of ARG1‐D, as determined by the presence of pathogenic *ARG1* variants or a deficiency in red blood cell ARG1 activity. Elevated plasma arginine was also permitted for an ARG1‐D diagnosis in PEACE. During the screening period, pARG levels were required to be > 200 μmol/L or ≥ 250 μmol/L for Phase 1/2 and PEACE, respectively. Subjects were excluded if they experienced hyperammonaemia (≥ 100 μmol/L) either requiring hospitalisation within the 14 days before enrolment (Phase 1/2) or requiring hospitalisation or emergency room management within the 6 weeks before the first dose of study treatment was administered (PEACE), or they had an active infection requiring treatment, or a known infection with human immunodeficiency virus (HIV), hepatitis B, or hepatitis C, or had any medical condition, comorbidity, or laboratory abnormality that, in the opinion of the Investigator, might have compromised the subject's safety, interfered with study participation or compliance, or affected the integrity or interpretation of study data (e.g., severe intellectual disability precluding required assessments). Subject enrolment for PEACE was limited to those with an existing deficit in any of the functional mobility assessments. However, subjects with extreme impairments rendering them unable to complete clinical assessments were excluded. The exclusion criteria also prevented those involved in previous pegzilarginase studies from participating in PEACE.

### 2.2 Biochemical Assessments

For both the Phase 1/2 study and PEACE, blood samples were drawn from each subject during the screening period, prior to treatment with pegzilarginase, and sent to a central laboratory for evaluation of plasma arginine. Plasma ammonia was analysed locally, and genotypes and red blood cell arginase activity were collected from historical determination, if available, or analysed centrally.

### 2.3 Clinical Characteristics

Baseline disease burden and clinical characteristics were determined prior to treatment with pegzilarginase, using a range of motor, cognitive, and quality of life (QoL) assessments. Data reported included the nature of initial symptoms, age of symptom onset, and age of first presentation of cognitive impairments, speech impairments, seizures, and muscle cramps. Symptom progression was assessed in individuals with three or more of these clinical presentations if age of onset data were available. Recent hospitalisations as well as the use of treatments and assistive devices were also assessed across the cohort.

The Gross Motor Function Classification System (GMFCS), as used for individuals with CP, was regarded as a suitable assessment to classify gross motor function, given the similarities in spasticity between CP and ARG1‐D [26]. The GMFCS categorises gross motor function based on the ability of subjects to complete self‐initiated and unaided movements. It comprises five levels which increase with the severity of impairment: Level 1 = minor motor deficits, Level 5 = severe impairments in motor function. The severity and location of spasticity, walking ability, and location of muscle cramps were also reported across the cohort.

Neurological assessments were carried out at baseline. The Full Scale IQ (FSIQ) was used to provide a combined summary report of subject intelligence, assessed by appropriately qualified professionals using the age‐dependent Weschler Intelligence Scales (WAIS‐IV, WISC‐V, or WPPSI‐IV) to ensure comparability between sites [27]. Possible IQ scores ranged from < 20–160, with 90–109 considered average. The presence of cognitive deficits, speech impairments, and history of seizures was also assessed.

QoL measures included the Paediatric QoL (PedsQL) Generic Core Scales, which assessed the physical, emotional, social, and school functioning of subjects aged 2 to 18 years [28]. PedsQL proxy data were obtained from parent‐ or caregiver‐proxy reports for all subjects within that age group. A reference dataset [29] used for comparison was also based on proxy reports. While PedsQL primarily captures functional ability rather than perceived burden, it remains a validated and widely used measure in paediatric chronic disorders. It was employed in this analysis for comparative interpretation in ARG1‐D.

### 2.4 Growth Indicators

Growth parameters were assessed at baseline in subjects aged 2–20 years. The height and weight measurements of subjects were plotted relative to an age‐ and sex‐matched normal population, using the National Center for Health Statistics' CDC Growth Charts Data Files [30].

### 2.5 Statistics and Generation of Figures

Descriptive statistics were used to summarise demographic and clinical characteristics. Graphical representations of the data were created using GraphPad Prism (Version 10.3.0).

QoL measures were compared with those reported for a reference population of typically developing children [29]. The magnitude of the impact of ARG1‐D was interpreted using the effect size framework [31]. The difference between the mean score of the reference population and the ARG1‐D population was calculated and divided by the standard deviation of the reference population, in line with Cohen's definition of effect size *d*. The resulting value was interpreted as indicating a small (≥ 0.20), medium (≥ 0.50), or large (≥ 0.80) effect, reflecting the degree to which ARG1‐D affected the QoL measure.

A sub‐group analysis was performed to explore the characteristics of patients identified through newborn screening (NBS) versus those diagnosed based on clinical presentation. Given the established relationship between early diagnosis and earlier dietary management in ARG1‐D, characteristics were also examined with respect to age at initiation of dietary protein restriction. Logistic regression analyses were conducted to examine the associations between NBS status, age at dietary intervention, age at baseline, and key clinical outcomes, and to evaluate the potential for confounding by age.

## 3 Results

### 3.1 Population Demographics

The demographics and baseline characteristics of the two study groups are summarised in Table 1. Across the collective cohort, the mean (SD) age at enrolment was 12.2 (7.4) years. Subjects were enrolled from a total of eight countries, and the overall proportions of males and females were equal. Details of the *ARG1* mutations within the population are presented in Table S1.

**TABLE 1:** Demographics of the study populations.

Patient information	Study 101A (*n* = 16)	PEACE (*n* = 32)	Pooled 101A + PEACE (*n* = 48)
Age at enrolment, y			
Mean ± SD	15.1 ± 8.5	10.7 ± 6.47	12.2 ± (7.4)
Median	15.0	10.5	11.5
Min, max	5, 31	2, 29	2, 31
Sex, *n* (%)			
Male	5 (31.3)	19 (59.4)	24 (50.0)
Female	11 (68.8)	13 (40.6)	24 (50.0)
Race, *n* (%)			
White/Caucasian descent	11 (68.8)	14 (43.8)	25 (52.1)
Asian	2 (12.5)	6 (18.8)	8 (16.7)
Black/African descent	1 (6.3)	2 (6.3)	3 (6.3)
Multiple race	0 (0)	2 (6.3)	2 (4.2)
Other	2 (12.5)	6 (18.8)	8 (16.7)
Missing	0 (0)	2 (6.3)	2 (4.2)
Region, *n* (%)			
US	10 (62.5)	14 (43.8)	24 (50.0)
Non‐US	6 (37.5)	18 (56.3)	24 (50.0)
Country, *n* (%)			
Austria	0 (0)	2 (6.3)	2 (4.2)
Canada	3 (18.8)	1 (3.1)	4 (8.3)
France	0 (0)	6 (18.8)	6 (12.5)
Germany	0 (0)	1 (3.1)	1 (2.1)
Italy	0 (0)	3 (9.4)	3 (6.3)
Portugal	2 (12.5)	0 (0)	2 (4.2)
United Kingdom	1 (6.3)	5 (15.6)	6 (12.5)
United States of America	10 (62.5)	14 (43.8)	24 (50.0)
Age at initial symptoms^a^, y			
Mean ± SD	2.7 ± 5.7	1.9 ± 2.4	2.2 ± 3.6
Median	0.7	1.0	1.0
Min, max	0, 19	0, 10	0, 19
Age at diagnosis^b^, y			
Mean ± SD	4.7 ± 7.4	3.3 ± 3.8	3.7 ± 5.0
Median	1.7	2.6	2.1
Min, max	0, 25	0, 15	0, 25

Abbreviations: *n*, number of subjects; SD, standard deviation; y, years.

^a^ Age at initial symptoms was available for 13 subjects in Study 101A and 32 in PEACE.

^b^ Age at diagnosis was available for 12 subjects in Study 101A and 32 in PEACE.

### 3.2 Onset of ARG1‐D

Across the ARG1‐D cohort, symptoms initially presented at a mean (SD) age of 2.2 (3.6) years (*n* = 45). The mean (SD) age of ARG1‐D diagnosis was 3.7 (5.0) years (*n* = 44).

### 3.3 Clinical and Biochemical Measures

Unlike other UCDs, where hyperammonaemia is often the primary biochemical finding, ARG1‐D is more characteristically marked by persistent hyperargininaemia. As stipulated by the inclusion criteria, all subjects exhibited elevated levels of plasma arginine during the screening period with mean (SD) plasma arginine of 392 (98) μmol/L and mean (SD) arginase activity in red blood cells of 2.2% (6.0%) (Table 2). Mean (SD) levels of plasma ammonia were 39 (24) μmol/L at baseline and 25/48 (52%) of subjects had a history of hyperammonaemic episodes.

**TABLE 2:** Clinical and biochemical measures.

Clinical and biochemical measures	Study 101A (*n* = 16)	PEACE (*n* = 32)	Pooled 101A + PEACE (*n* = 48)
Baseline plasma arginine, μmol/L			
Mean ± SD	373 ± 91	402 ± 102	392 ± 98
Median	389	398	395
Min, max	238, 566	202, 573	202, 573
Within normal range, *n* (%)	0 (0)	0 (0)	0 (0)
Arginase activity in RBCs, %			
*n*	16	30^a^	46
Mean ± SD	2.3 ± 4.9	2.2 ± 6.5	2.2 ± 6.0
Median	0.1	0.0	0.0
Min, max	0, 15	0, 25	0, 25
Liver test abnormality, *n* (%)			
ALT/AST elevated	9 (56.3)	22 (68.8)	31 (64.6)
Prothrombin time/INR elevated	2 (12.5)	11 (34.4)	13 (27.1)
Abnormal fibrinogen	0 (0)	0 (0)	0 (0)
Gamma‐glutamyl transferase elevated	0 (0)	2 (6.3)	2 (4.3)
Other	2 (12.5)	1 (3.1)	3 (6.3)
Baseline plasma ammonia, μmol/L			
Mean ± SD	41.1 ± 21.5	37.6 ± 25.5	38.8 ± 24.0
Median	38.0	35.0	37.0
Min, max	9.0–77.0	9.0, 136.0	9.0, 136.0
History of hyperammonaemic episodes, *n* (%)			
Yes	7 (43.8)	18 (56.3)	25 (52.1)
No	9 (56.3)	14 (43.8)	23 (47.9)

Abbreviations: ALT, alanine aminotransferase; AST, aspartate aminotransferase; INR, international normalised ratio; *n*, number of subjects; RBC, red blood cells; SD, standard deviation.

^a^ Values were originally reported in μM/h/mg Hb. Of the 30 subjects, 26 had measurements of 0 μM/h/mg Hb, and four subjects had measurements of 0.1, 0.2, 0.5, and 0.5 μM/h/mg Hb, respectively. The lower limit of the normal range (2–7 μM/h/mg Hb) was used to calculate percentage activity.

**FIGURE 1:**
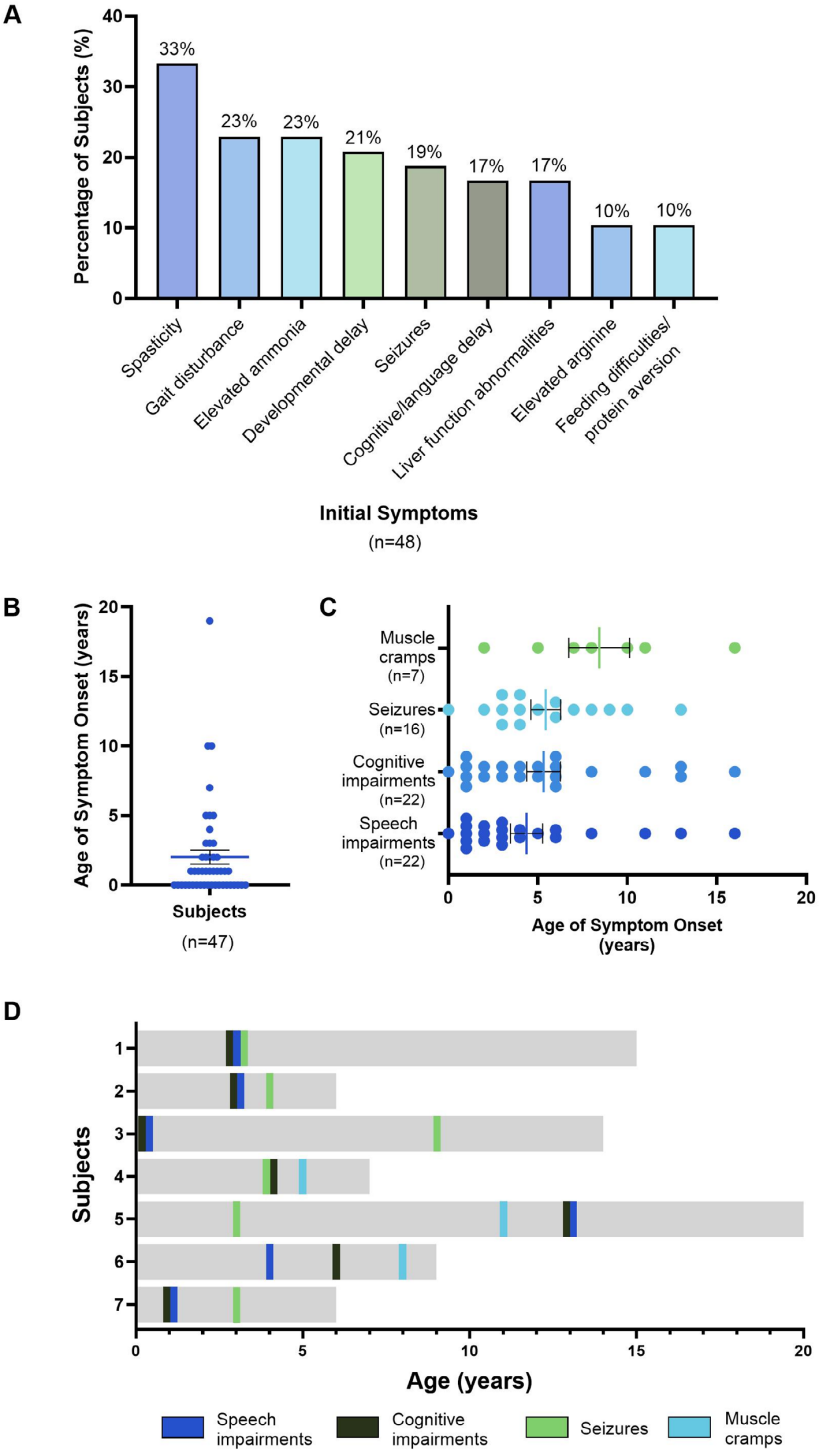
Nature and onset of ARG1‐D manifestations. Data were taken from individual patient narratives featuring rounded ages. (A) Initial ARG1‐D symptoms that subjects first presented with. Initial symptoms reported have been limited to those which occurred in > 10% of subjects. (B) Age of onset of ARG1‐D. (C) Age at which muscle cramps, seizures, cognitive and speech impairments first presented. For (B) and (C), each point represents one individual. Both show mean with SEM. (D) The progression of symptoms, using age of symptoms manifested, in individuals with at least three of the four assessed symptoms. Grey bars indicate subject age.

Spasticity was the most common initial ARG1‐D symptom presented, reported in one‐third of subjects (16/48, 33%), followed by gait disturbances (11/48, 23%), elevated ammonia (11/48, 23%), and developmental delay (10/48, 21%) (Figure 1). Although data regarding the age at which spasticity presented were not available, the age of onset of cognitive and speech impairments, seizures, and muscle cramps was assessed. Speech impairments typically presented earliest, at a mean (SD) age of 4.4 (4.2) years, followed by cognitive impairments (5.3 [4.4] years) and seizures (5.4 [3.3] years). To understand the progression of the disease, the age at which these symptoms appeared was assessed in subjects who had presented with at least three out of the four symptoms (*n* = 7). Symptoms developed over time. In 5/7 (71%) subjects, cognitive and speech impairments presented together at the same age. No other clear patterns in the order of symptom progression were observed.

### 3.4 Neuromotor Function Characteristics

**FIGURE 2:**
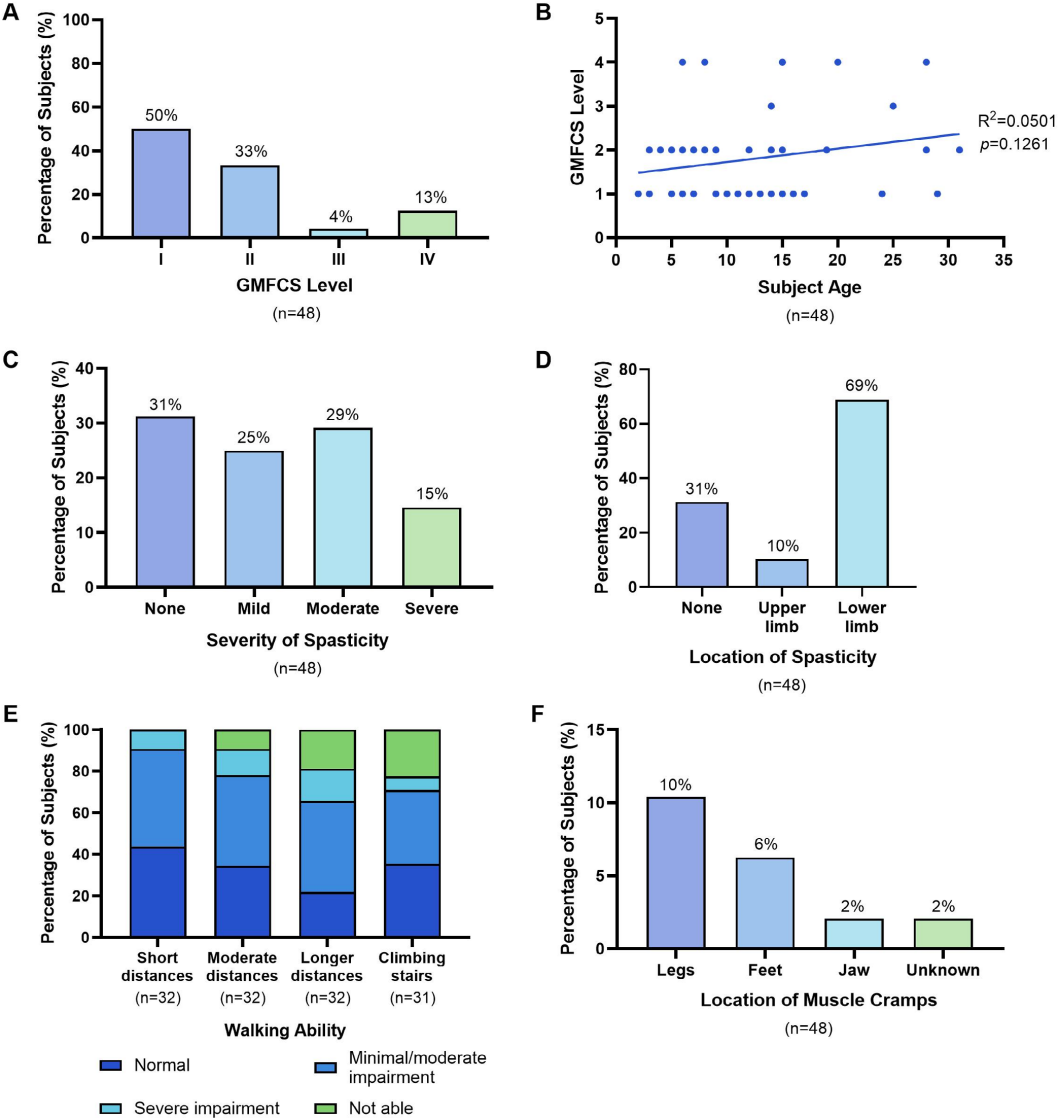
Neuromotor clinical characteristics of ARG1‐D. (A) GMFCS level of the ARG1‐D cohort. (B) GMFCS level of subjects relative to subject age. Each dot represents one individual. Simple linear regression with *R* ^2^ and *p* value indicated. (C) Percentage of subjects with spasticity and indication of severity. (D) Location of spasticity. Of the 33 subjects exhibiting spasticity in the lower limbs, five also exhibited spasticity within the upper limbs. (E) Walking ability of subjects across short (5 m), medium (50 m), and longer distances (500 m), and when climbing stairs. Data from subjects in PEACE study only. One subject's ability to climb stairs was unknown. (F) Location of muscle cramps.

Clinical characteristics related to neuromotor deficits are presented in Figure 2. The mobility and gross motor skills of all subjects were assessed using the GMFCS. All subjects across the cohort had a GMFCS Level I or higher, indicative of difficulties with speed, balance, and coordination. Of these subjects, 16/48 (33%) were GMFCS Level II, suggesting difficulties walking on uneven surfaces or inclines, climbing stairs unassisted, and minimal ability to run or jump, while 2/48 (4%) and 6/48 (13%) were Levels III and IV, respectively, which represent severe deficits in gross motor function and indicate a requirement for assistive mobility devices for standing, walking, and climbing stairs. These differences in mobility across the cohort were reflected in the assessment of walking ability. Overall, the level of impairment in walking ability increased with longer distances and when climbing stairs. Interestingly, the GMFCS level of subjects was not found to significantly correlate with age (*R* ^2^ = 0.05, *p* = 0.13).

Spasticity, a hallmark characteristic of ARG1‐D, was present in 33/48 (69%) of subjects, and was categorised as mild, moderate, or severe in 12/48 (25%), 14/48 (29%), and 7/48 (15%) of subjects, respectively. All subjects exhibiting spasticity reported involvement of the lower limbs, and 5/48 (10%) also exhibited spasticity within the upper limbs. Overall, 8/48 (17%) subjects experienced muscle cramps, of which cramps in the legs were most common.

### 3.5 Neurological Clinical Characteristics

**FIGURE 3:**
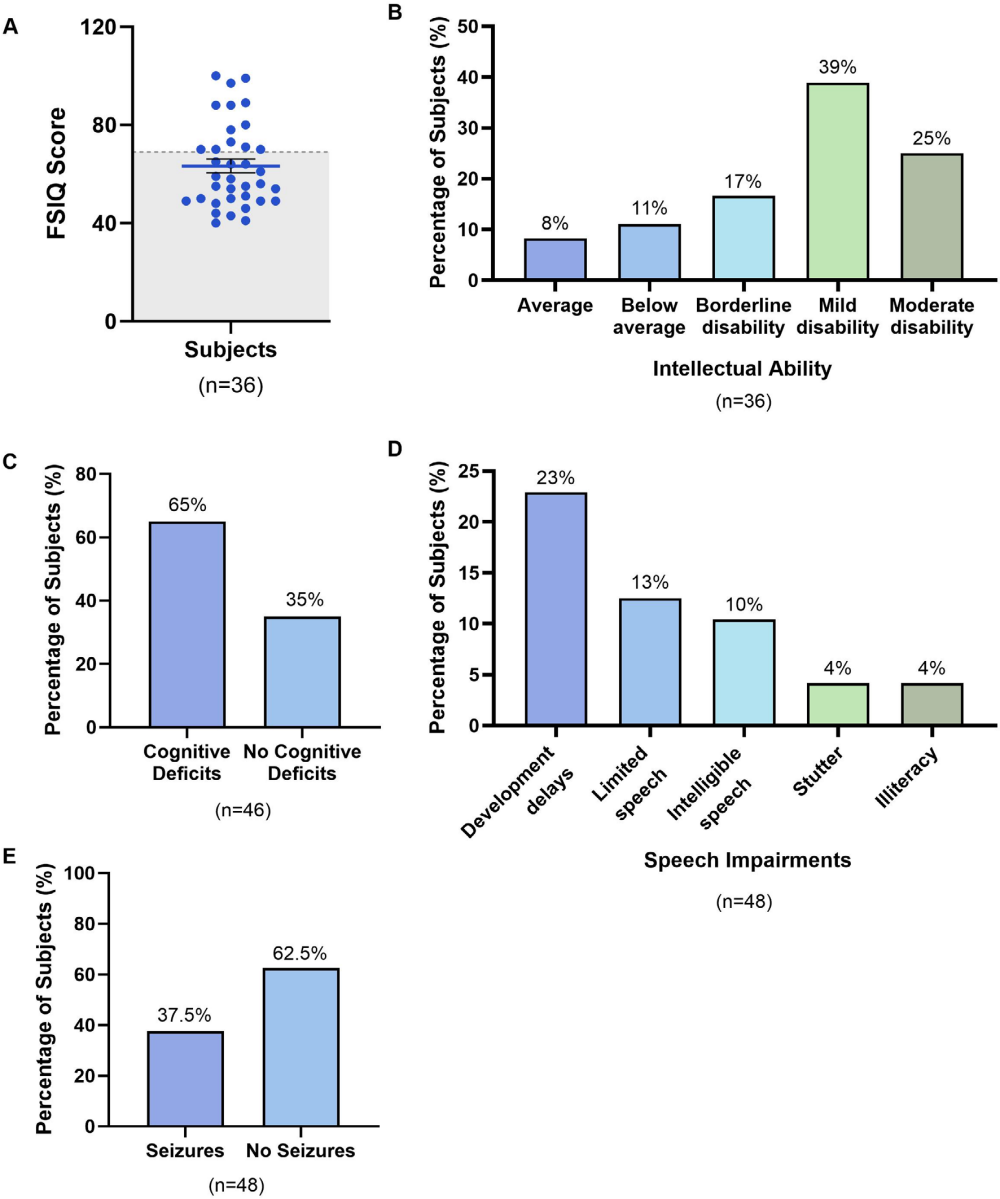
Neurological clinical characteristics of ARG1‐D. (A, B) Intellectual ability of subjects, as determined by FSIQ score, using Wechsler's classification. In (A), grey shading indicates FSIQ scores associated with intellectual disability (≤ 69). In (B), Average = FSIQ scores of 90–109, below average = 80–89, borderline = 70–79, mild disability = 50–69, and moderate disability = 35–49. (C–E) Percentage of subjects with cognitive deficits (C), speech impairments (D), and history of seizures (E).

To understand the clinical neurological characteristics of individuals with ARG1‐D, assessments of intellectual ability, cognitive deficits, speech impairments, and seizures were undertaken at baseline (Figure 3). FSIQ scores were used to determine the intellectual ability of subjects. Overall, 23/36 (64%) of subjects scored lower than 69, indicative of intellectual disability, of which 14/36 (39%) and 9/36 (25%) were classified as having a mild and moderate disability, respectively. Cognitive deficits were present in 30/46 (65%) subjects and 26/48 (54%) exhibited speech and language deficits. Of the latter, developmental delays were most common, present in 11/48 (23%) of individuals, followed by limited and intelligible speech. Seizures were also a prominent neurological characteristic of the ARG1‐D cohort and were reported in 18/48 (38%) of individuals.

### 3.6 Quality of Life (PedsQL)

The PedsQL Generic Core Scales were used to assess the QoL of subjects aged 2 to 18 years. Across the ARG1‐D cohort, physical and psychosocial functioning were severely affected, with mean (SD) proxy scores of 68 (26) and 64 (16) out of a possible score of 100, compared with the mean proxy scores for a population of typically developing children of 85 (20) and 82 (15) (Table 3). ARG1‐D had a large effect on QoL.

**TABLE 3:** Parent‐reported quality of life measures.

Parent‐reported quality of life assessments (PedsQL)^a^	Study 101A	PEACE	Pooled 101A + PEACE	Typically developing reference population^b^	Effect size^c^
Total score	66.8 ± 6.9	65.6 ± 18.2	65.7 ± 17.5	82.7 ± 15.4	Large (1.10)
Mean ± SD, *n*	*n* = 2	*n* = 23	*n* = 25	*n* = 9430	
Physical	73.4 ± 11.1	67.1 ± 26.8	67.6 ± 25.8	84.5 ± 19.5	Large (0.84)
Mean ± SD, *n*	*n* = 2	*n* = 23	*n* = 25	*n* = 9413	
Psychosocial	63.3 ± 4.7	64.5 ± 16.5	64.4 ± 15.9	81.7 ± 15.2	Large (1.13)
Mean ± SD, *n*	*n* = 2	*n* = 23	*n* = 25	*n* = 9431	

Abbreviations: *n*, number; PedsQL, paediatric quality of life; SD, standard deviation.

^a^ The PedsQL assessment in 101A and PEACE was undertaken for subjects aged 2–18 years. Its scales comprise areas with a potential score range from 0 to 100, where greater scores indicate a higher quality of life.

^b^ The typically developing reference population was derived from the PedsQL 4.0 initial field test and a statewide State Children's Health Insurance Program evaluation reported by Varni et al. [29].

^c^ Effect size was calculated by taking the difference between the reference population mean and the Pooled 101A + PEACE sample mean, divided by the reference population standard deviation. Effect sizes for differences in means are designated as small (0.20), medium (0.50), and large (0.80) in magnitude [31].

### 3.7 Growth Indicators

**FIGURE 4:**
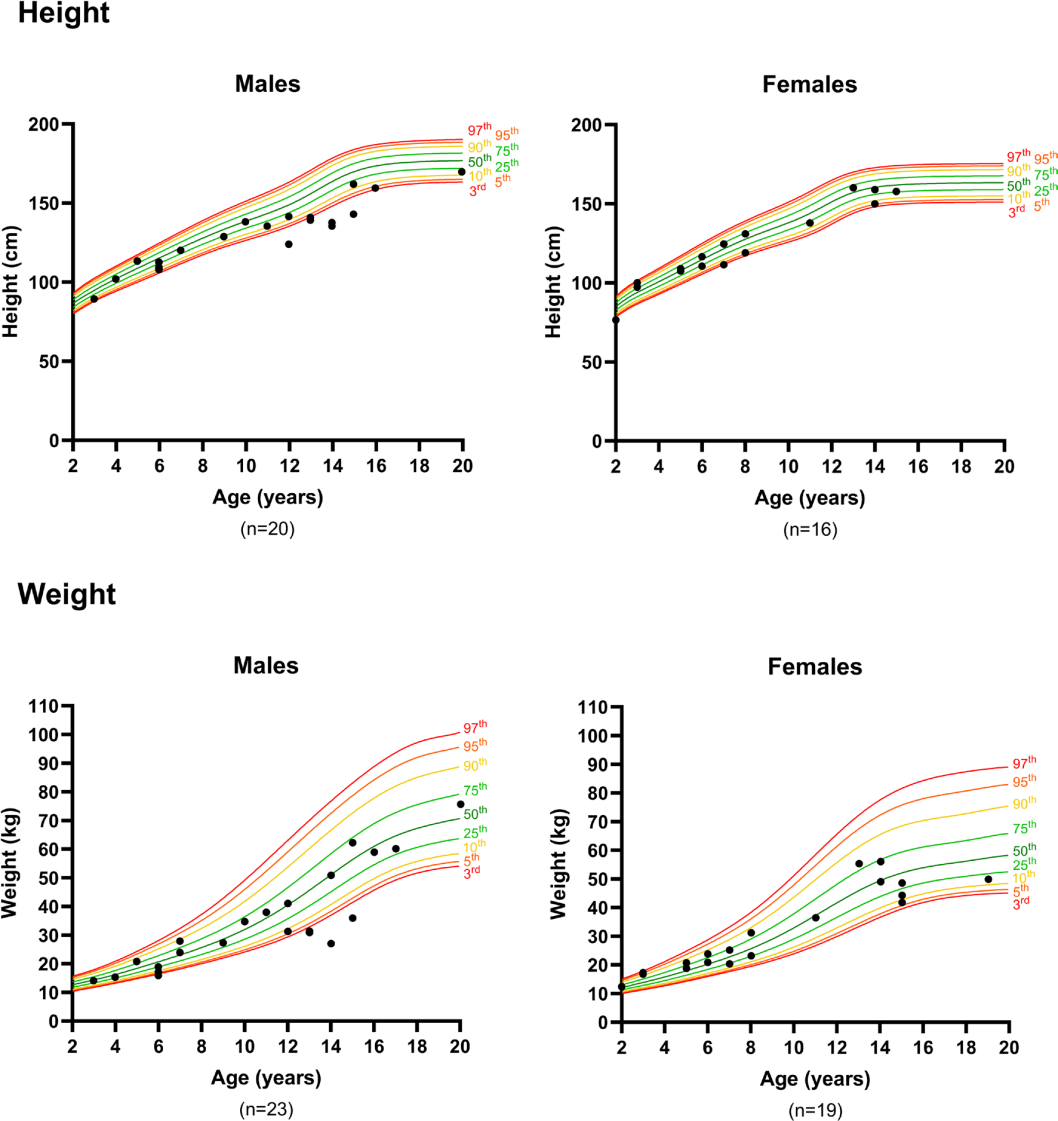
Growth indicators in the ARG1‐D cohort. Height and weight of individuals aged 2 to 20 years in the ARG1‐D cohort, relative to an age‐ and sex‐matched normal population. Growth charts generated using the National Centre for Health Statistics' CDC Growth Charts Data Files.

The height and weight of subjects aged 2–20 years were plotted relative to an age‐ and sex‐matched normal population (Figure 4). Typically, measurements between the 3rd and 97th percentile are considered normal [32]. The height and weight distribution of male subjects was more varied across the ARG1‐D cohort than for females. Of note, for the males, 6/20 (30%) were below the 3rd percentile for height and 5/23 (22%) for weight, whereas only one female (1/16, 6%) was below the 3rd percentile for height. Whilst measurements below the 3rd percentile can indicate poor growth, firm conclusions cannot be drawn without longitudinal data.

### 3.8 Use of Treatments and Assistive Devices

**FIGURE 5:**
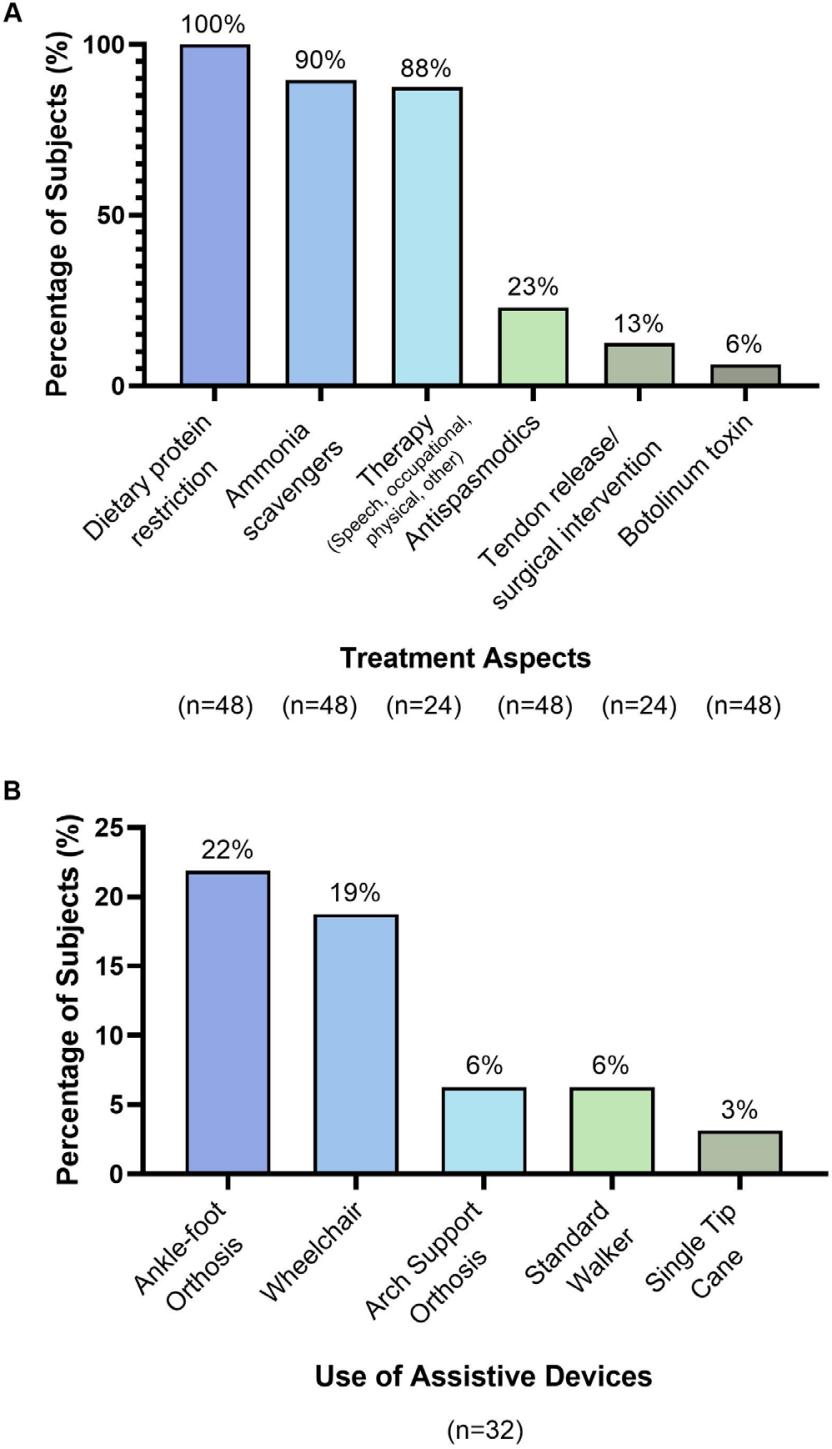
Treatment aspects and use of assistive devices across the ARG1‐D cohort. (A) Percentage of subjects using different treatments as part of standard‐of‐care regimens, across the ARG1‐D cohort. Data was not complete for all subjects. (B) Percentage of subjects using assistive devices across the cohort. Overall, 12/32 (38%) used assistive devices. Data was only available from subjects in PEACE.

Standard of care treatment regimens, the use of assistive devices, and the rate of hospitalisations were also assessed across the ARG1‐D cohort (Figure 5). All patients (48/48, 100%) had restrictions on dietary protein as part of their standard of care treatment regimens, and nitrogen scavengers were used by 43/48 (90%) of individuals. Therapy, comprising speech, occupational, physical, and other types of therapy, was used by 21/24 (88%) of individuals. Use of antispasmodics, tendon release, surgical intervention, and botulinum toxin was less common. Assistive devices were used by 12/32 (38%) of subjects, the most common of which were ankle‐foot orthosis and wheelchairs. A total of 17/48 (35%) of subjects were hospitalised within the 2 years prior to study initiation, with a mean (SD) of 2.4 (3.0) hospitalisations during this period. However, it is important to note that for the PEACE study group, hospitalisations only referred to non‐hyperammonaemic hospitalisations.

### 3.9 Possible Effects of Early Identification and Treatment Intervention

Patients identified through NBS had fewer and/or milder clinical manifestations of the disease as measured by GMFCS level, spasticity, muscle cramps, seizures, walking abilities, and degree of delay in cognition and language (Table S2). There was the potential for confounding, given that subjects identified through NBS (and therefore with earlier dietary intervention) were on average younger at baseline than those diagnosed based on clinical symptoms. Logistic regressions were performed to further assess the relationships (data not shown). Adjustment for age at baseline did not materially alter the associations observed, indicating that age at baseline did not correlate with clinical outcomes at baseline, but NBS status and age at dietary intervention did.

## 4 Discussion

This study provides the most comprehensive cross‐sectional characterisation of ARG1‐D to date, drawing on harmonised baseline data from international trials. In addition to confirming some expected features already reported in the literature, the study quantifies neuromotor, cognitive, and quality‐of‐life outcomes using standardised instruments.

*ARG1* variations presented across the cohort broadly reflected those reported by Diez‐Fernandez et al. [33]. As expected from the inclusion criteria, all subjects had elevated plasma arginine and impaired mobility, with lower limb spasticity present in most. Symptom onset is typically between 2 and 4 years of age; ARG1‐D rarely presents in the neonatal period [4]. The mean age at onset in this cohort (2.2 years) preceded the mean age of diagnosis (3.7 years). Delays in diagnosis, as reported elsewhere [10] highlight continued under‐recognition and frequent misdiagnosis as CP or hereditary spastic paraplegia [5, 6, 7].

The biochemical profile observed aligns with the established natural history of ARG1‐D, with consistently elevated plasma arginine and reduced red cell arginase activity. Hyperargininaemia and elevated levels of guanidino compounds (GCs) were a consistent finding, corroborating previous biochemical reports of ARG1‐D in which levels were raised in both the blood and cerebrospinal fluid [34, 35, 36]. GCs have been mechanistically associated with the induction of hyperexcitability, excitotoxicity, and oxidative stress, the deleterious effects of which become evident in the first years of life and cumulatively increase in severity throughout the patient journey [4, 8, 9, 10, 15, 37, 38]. As such, GCs are thought to have a prominent role in the neurological manifestations of ARG1‐D, including spasticity, intellectual impairments, and developmental delays. Given that GCs are metabolites of arginine, arginine is implicated as a key driver of neurological pathology.

Although hyperammonaemia is not a dominant feature of ARG1‐D, more than half of subjects reported historical episodes consistent with recent natural‐history studies reporting hyperammonaemic episodes in ARG1‐D patients. Two recent studies reported hyperammonaemia‐related deaths, suggesting this risk may be significantly under‐appreciated [10, 39].

The systematic baseline assessments illustrate the heterogeneity of neuromotor and cognitive impairment. Use of the GMFCS, FSIQ and PedsQL instruments enabled consistent quantification of function across sites and age groups. Spasticity was present in 69% of subjects and, as in previous literature, primarily affected the lower limbs. Severity varied independently of age, demonstrating that functional status is not solely age‐dependent but reflects intrinsic disease heterogeneity.

Cognitive and language deficits were common (65% and 54% respectively) and intellectual disability was present in nearly two‐thirds of assessed subjects. Throughout the literature, memory, attention and problem‐solving deficits, hyperactivity, and emotional instability, have been reported [15]. Magnetic resonance imaging has revealed insights into the underlying brain pathology associated with ARG1‐D, with dysmyelination, cerebral cortical atrophy, corticospinal tract abnormalities, and alterations in the structural integrity of white matter amongst the most commonly reported phenotypes [12, 14, 15, 40] revealing the extent of central nervous system involvement in ARG1‐D pathology.

All patients in this cohort exhibited some degree of motor dysfunction, likely limiting independence and daily activities. Cognitive impairments may have had implications for social interactions, education, and emotional well‐being. A study in the USA comparing QoL of children with normal development versus a group with CP [29] found that the PedsQL proxy scores of the CP group were around one‐third lower than those of children with normal development. By indirect comparison, the PedsQL proxy scores of our cohort were around 20% lower than those children with normal development. As with CP, ARG1‐D appears to have a large and statistically detrimental effect on QoL, and it is likely to be more pronounced than we have described, given that patients with the most severe disease state were not included in the clinical studies. Additionally, unlike CP, ARG1‐D is a progressive disorder with deficits across multiple domains emerging over time, suggesting that QoL could be expected to worsen as the disease advances. Separate to the impairments in functional abilities measured by PedsQL, ARG1‐D patients and their caregivers must also manage the ongoing challenges of dietary management including adherence to protein restriction and reliance on specialised foods. Beyond the burden on patients and caregivers, ARG1‐D has also been shown to create significant healthcare and wider socioeconomic costs [41].

As indicated in longitudinal studies, ARG1‐D is progressive in nature. Accordingly, earlier diagnosis to facilitate timely treatment intervention may be key to limiting neuromotor and neurocognitive impairments and improve patient outcomes [13]. Diagnosis of ARG1‐D can be achieved via routine amino acid testing, confirmed by genetic or red blood cell enzyme activity analysis. Increased awareness and broader use of diagnostic screening—including NBS—could support earlier detection. Our analyses comparing subjects identified via NBS with early treatment intervention versus those diagnosed based on clinical symptoms with later treatment intervention may reflect the value of early detection and treatment initiation in preventing/mitigating disease symptoms. However, the cross‐sectional design of our study precludes us from making firm claims regarding causality.

Our study has several limitations: its cross‐sectional design limits longitudinal inference, enrolment criteria introduce selection bias toward subjects with preserved, albeit substantially impaired, function, and the absence of untreated controls limits comparison. Additionally, for PedsQL, cross‐national differences in expectations for child functioning may affect scoring, and proxy ratings may reflect observable functional limitations but underestimate subjective emotional well‐being—particularly given the challenges of dietary management and adherence to protein restriction and specialised foods. Nonetheless, we contend that these harmonised baseline data provide valuable insights into disease burden across a large internationally diverse cohort. Overall, the findings demonstrate that ARG1‐D imposes a substantial functional and psychosocial burden, despite standard care.

## 5 Conclusion

ARG1‐D is characterised by its insidious onset and progressively debilitating nature. It presents a considerable disease burden for individuals with the disease. While lower‐limb spasticity related to chronic hyperargininaemia is the dominant disease characteristic, a heterogenous array of neurological and neuromotor phenotypes are present throughout the affected population. Timely diagnosis and optimised management remain the key clinical approaches to mitigating disease burden and increasing QoL for individuals with ARG1‐D.

## Author Contributions

Nancy Braverman, Richard Chang, Gregory M. Enns, Arunabha Ghosh, Magali Gorce, Daniela Karall, Reena Sharma, Emily Shelkowitz, Roberto Zori, and Markey McNutt contributed to data collection, data interpretation, and writing and review of the manuscript. Mattias Rudebeck contributed to data analysis, data interpretation, literature search, and writing and review of the manuscript. All authors approved the final manuscript.

## Funding

Immedica Pharma AB funded the medical writing of this article. Aeglea BioTherapeutics Inc. funded the clinical studies described.

## Ethics Statement

The studies described were conducted in accordance with the International Council for Harmonisation of Technical Requirements for Pharmaceuticals for Human Use (ICH) E6 guidelines for Good Clinical Practice (GCP), the Declaration of Helsinki, and IRB or IEC requirements. As applicable, the studies were also conducted in accordance with the United States (US) Food and Drug Administration (FDA) regulations, EU Clinical Trials Directive 2001/20/EC, the United Kingdom (UK) Medicines for Human Use (Clinical Trials) Regulations 2004, and Canada Drug‐Part C, Division 5 of the Food and Drugs Act and Regulation, and all other applicable local and national laws and regulations governing the conduct of human clinical studies.

## Consent

Written informed consent was obtained from all subjects or their guardians or legal representatives, as applicable for local laws.

## Conflicts of Interest

Mattias Rudebeck is an employee and shareholder of Immedica Pharma AB. The other co‐authors were investigators in the pegzilarginase trial programme. Additionally, Gregory Enns has received research funding from Immedica Pharma AB, Arunabha Ghosh has received consultancy fees from Immedica Pharma AB, Magali Gorce has received consultancy fees and honoraria from Immedica Pharma AB for educational activities and patient support tools, Daniela Karall and Reena Sharma have received consultancy fees and honoraria from Immedica Pharma AB, Roberto Zori has received travel support from Aeglea Biotherapeutics, and Markey McNutt has received funding, honoraria, and travel support from Horizon Therapeutics, Arcturus Therapeutics, and Aeglea Biotherapeutics.

## Supporting information


**Table S1:** jimd70156‐sup‐0001‐TableS1.docx. *ARG1* mutations presented across the cohorts.

**Table S2:** Comparison of baseline demographic and clinical characteristics in patients identified by newborn screening versus later clinical presentation and age of dietary intervention.

## Data Availability

Data access will be granted in response to qualified collaborative research requests. Aggregate study data can be made available to researchers. Data will be shared based on the basis of the scientific merit of the proposal (i.e., the proposal should be scientifically sound, ethical, and have the potential to contribute to the advancement of public health) and the feasibility of the collaborative research proposal (i.e., the requesting research team must be scientifically qualified and have the resources for the proposed project). The data files would exclude data dictionaries that require user licenses. Data could be made available following finalised regulatory authority reviews and at the end of any data exclusivity periods and ending 24 months after the regulatory authority review decision has been received or until the corresponding author is able to fulfil this obligation, whichever is earlier. Furthermore, the study protocol and statistical analysis plan can at this time be made available. Proposals should be directed to the corresponding author. Data requestors will need to sign a data access agreement.
