# Supplementary material for: Clinical Characteristics of Arginase 1 Deficiency: Natural History Insights From International Clinical Trials
Source: J Inherit Metab Dis. 2026 Feb 6;49(2):e70156. doi: 10.1002/jimd.70156 (PMC12880898; doi:10.1002/jimd.70156)
Supplement: Supplementary file 1 — Table S1: jimd70156‐sup‐0001‐TableS1.docx. ARG1 mutations presented across the cohorts. [file JIMD-49-0-s001.docx]

**Table S1:** ***ARG1* mutations presented across the cohorts**

| **Variant(s)** | **Variant Type(s) and Length(s)** | **Variant Molecular Consequence(s)** | **Zygosity** | **Number (%) of Subjects** |
| --- | --- | --- | --- | --- |
| NM_000045.4(ARG1):c.466-1G>C (p.?) | single nucleotide variant, 1 bp | splice acceptor | Homozygous (6) | 6 (12.5%) |
| NM_000045.4(ARG1):c.23T>A (p.Ile8Lys) | single nucleotide variant, 1 bp | missense | Homozygous (1), Heterozygous (1), Unknown (1) | 3 (6.3%) |
| NM_000045.4(ARG1):c.314_345delinsATGCAAGCATCAGGATGACT (p.Ile105_His115delinsAsnAlaSerIleArgMetThr) | indel, 32 bp | inframe indel | Homozygous (1), Unknown (2) | 3 (6.3%) |
| NM_000045.4(ARG1):c.61C>T (p.Arg21Ter) | single nucleotide variant, 1 bp | nonsense | Homozygous (2), Heterozygous (1) | 3 (6.3%) |
| NM_000045.4(ARG1):c.370G>T (p.Asp124Tyr) | single nucleotide variant, 1 bp | missense | Homozygous | 2 (4.2%) |
| NM_000045.4(ARG1):c.749G>A (p.Gly250Glu) | single nucleotide variant, 1 bp | missense | Homozygous | 2 (4.2%) |
| NM_000045.4(ARG1):c.849delG (p.Lys284Argfs*6) | deletion, 1 bp | frameshift | Homozygous | 2 (4.2%) |
| NM_000045.4(ARG1):c.93del (p.Arg32fs) | deletion, 1 bp | frameshift | Homozygous | 2 (4.2%) |
| NM_000045.4(ARG1):c.130+1G>A (p.?) | single nucleotide variant, 1 bp | splice donor | Homozygous | 1 (2.1%) |
| NM_000045.4(ARG1):c.23T>G (p.Ile8Arg) | single nucleotide variant, 1 bp | missense | Homozygous | 1 (2.1%) |
| NM_000045.4(ARG1):c.272dup (p.Arg92fs) | duplication, 1 bp | frameshift | Homozygous | 1 (2.1%) |
| NM_000045.4(ARG1):c.314_345delinsATGCAAGCATCAGGATGACT (p.Ile105_His115delinsAsnAlaSerIleArgMetThr); NM_000045.4(ARG1):c.466-1G>C (p.?) | indel, 32 bp; single nucleotide variant, 1 bp | inframe indel; splice acceptor | Compound heterozygous | 1 (2.1%) |
| NM_000045.4(ARG1):c.3G>A (p.Met1Ile); NM_000045.4(ARG1):c.92T>G (p.Leu31Arg) | single nucleotide variant, 1 bp; single nucleotide variant, 1 bp | missense initiator codon variant; missense | Compound heterozygous | 1 (2.1%) |
| NM_000045.4(ARG1):c.449G>A (p.Gly150Glu) | single nucleotide variant, 1 bp | missense | Homozygous | 1 (2.1%) |
| NM_000045.4(ARG1):c.466-1G>C (p.?); NM_000045.4(ARG1):c.603_604delTG (p.Glu202Argfs*6) | single nucleotide variant, 1 bp; deletion, 2 bp | splice acceptor; frameshift | Compound heterozygous | 1 (2.1%) |
| NM_000045.4(ARG1):c.466-1G>C (p.?); NM_000045.4(ARG1):c.61C>T (p.Arg21Ter) | single nucleotide variant, 1 bp; single nucleotide variant, 1 bp | splice acceptor; nonsense | Compound heterozygous | 1 (2.1%) |
| NM_000045.4(ARG1):c.466-1G>C (p.?); NM_000045.4(ARG1):c.787G>T (p.Glu263Ter) | single nucleotide variant, 1 bp; single nucleotide variant, 1 bp | splice acceptor; nonsense | Compound heterozygous | 1 (2.1%) |
| NM_000045.4(ARG1):c.532G>C (p.Gly178Arg) | single nucleotide variant, 1 bp | missense | Homozygous | 1 (2.1%) |
| NM_000045.4(ARG1):c.561-2A>T (p.?) | single nucleotide variant, 1 bp | splice acceptor | Homozygous | 1 (2.1%) |
| NM_000045.4(ARG1):c.57+1G>A (p.?); NM_000045.4(ARG1):c.151G>T (p.Gly51Cys) | single nucleotide variant, 1 bp; single nucleotide variant, 1 bp | splice donor; missense | Compound heterozygous | 1 (2.1%) |
| NM_000045.4(ARG1):c.646_649del (p.Leu216fs) | deletion, 4 bp | frameshift | Homozygous | 1 (2.1%) |
| NM_000045.4(ARG1):c.647ins32 (p.?); NM_000045.4(ARG1):c.871C>T (p.Arg291Ter) | insertion, 32 bp; single nucleotide variant, 1 bp | frameshift; nonsense | Compound heterozygous | 1 (2.1%) |
| NM_000045.4(ARG1):c.684delT (p.Leu229Ter); NM_000045.4(ARG1):c.683A>G (p.His228Arg) | deletion, 1bp; single nucleotide variant, 1 bp | nonsense; missense | Compound heterozygous | 1 (2.1%) |
| NM_000045.4(ARG1):c.695A>T (p.Asp232Val) | single nucleotide variant, 1 bp | missense | Homozygous | 1 (2.1%) |
| NM_000045.4(ARG1):c.700G>C (p.Asp234His) | single nucleotide variant, 1 bp | missense | Homozygous | 1 (2.1%) |
| NM_000045.4(ARG1):c.703_707delinsAGACTGGACC (p.Asp237Glufs*?) | indel, 5 bp | frameshift | Homozygous | 1 (2.1%) |
| NM_000045.4(ARG1):c.703G>A (p.Gly235Arg); NM_000045.4(ARG1):c.712_713insGGACC (p.Gly238fs) | single nucleotide variant, 1 bp; insertion, 5 bp | missense; frameshift | Compound heterozygous | 1 (2.1%) |
| NM_000045.4(ARG1):c.769G>C (p.Gly257Arg) | single nucleotide variant, 1 bp | missense | Homozygous | 1 (2.1%) |
| NM_000045.4(ARG1):c.802+2T>G (p.?) | single nucleotide variant, 1 bp | splice donor | Homozygous | 1 (2.1%) |
| NM_000045.4(ARG1):c.807_811delACTCT (p.Leu270Cysfs*3); NM_000045.4(ARG1):c.611A>G (p.Tyr204Cys) | deletion, 5bp; single nucleotide variant, 1 bp | frameshift; missense | Compound heterozygous | 1 (2.1%) |
| NM_000045.4(ARG1):c.888delA (p.Val297Leufs*3) | deletion, 1 bp | frameshift | Homozygous | 1 (2.1%) |
| NM_000045.4(ARG1):c.892G>C (p.Ala298Pro) | single nucleotide variant, 1 bp | missense | Homozygous | 1 (2.1%) |
| Not tested | Not tested | Not tested | Not tested | 1 (2.1%) |
| **Total** |  |  |  | **48 (100%)** |

All variants are reported using the reference transcript NM_000045.4, and classified according to their predicted molecular consequence and zygosity. Variant descriptions follow Human Genome Variation Society (HGVS) nomenclature. Variant type, length, and consequence were determined using ClinVar and manual review. Zygosity was classified based on the genetic test results reported in the clinical study data. Compound heterozygous was assigned when two distinct variants were identified in the same individual and confirmed or presumed to be on opposite alleles. The term Unknown was used when zygosity could not be determined due to incomplete or ambiguous source data.

One subject was not tested but their sibling has a homozygous mutation in the ARG1 gene c.802+T>G, and it is likely that the mutation in this subject is the same.
