# Supplementary material for: Clinical Characteristics of Arginase 1 Deficiency: Natural History Insights From International Clinical Trials
Source: J Inherit Metab Dis. 2026 Feb 6;49(2):e70156. doi: 10.1002/jimd.70156 (PMC12880898; doi:10.1002/jimd.70156)
Supplement: Supplementary file 2 — Table S2: Comparison of baseline demographic and clinical characteristics in patients identified by newborn screening versus later clinical presentation and age of dietary intervention. [file JIMD-49-0-s002.docx]

**Table S2: Comparison of baseline demographic and clinical characteristics in patients identified by newborn screening versus later clinical presentation and age of dietary intervention**

|  |  | **NBS^1^** | **Non-NBS** | **Early dietary restrictions^2^** | **Late dietary restrictions^2^** |
| --- | --- | --- | --- | --- | --- |
| **Age (years)** | n | 12 | 20 | 10 | 18 |
|  | Mean (SD) | 7.3 (4.1) | 12.8 (6.8) | 8.4 (4.5) | 11.1 (6.7) |
|  | Median | 7.5 | 13.5 | 9.5 | 10.0 |
|  | Min, Max | 2, 13 | 5, 29 | 2, 14 | 3, 29 |
| **Sex, n (%)** | Female | 5 (41.7) | 8 (40.0) | 5 (50.0) | 6 (33.3) |
|  | Male | 7 (58.3) | 12 (60.0) | 5 (50.0) | 12 (66.7) |
| **GMFCS, n (%)** | I | 9 (75.0) | 5 (25.0) | 7 (70.0) | 4 (22.2) |
|  | II | 3 (25.0) | 10 (50.0) | 3 (30.0) | 10 (55.6) |
|  | IV | 0 (0) | 5 (25.0) |  | 4 (22.2) |
| Analysis of GMFCS category I vs >I |  | p=0.0100 | | p=0.0204 | |
| **Level of Spasticity, n (%)** |  |  |  |  |  |
|  | None | 7 (58.3) | 4 (20.0) | 7 (70.0) | 4 (22.2) |
|  | Mild | 3 (25.0) | 6 (30.0) | 3 (30.0) | 4 (22.2) |
|  | Moderate | 2 (16.7) | 7 (35.0) | 0 (0) | 8 (44.4) |
|  | Severe | 0 (0) | 3 (15.0) | 0 (0) | 2 (11.1) |
| Analysis of ‘None’ vs ‘Mild/Moderate/Severe’ |  | p=0.0529 | | p=0.0204 | |
| **Muscle Cramps, n (%)** | No | 11 (91.7) | 14 (70.0) | 10 (100.0) | 13 (72.2) |
|  | Yes | 1 (8.3) | 6 (30.0) | 0 (0) | 5 (27.8) |
| Analysis of ‘No’ vs ‘Yes’ |  | p=0.2117 | | p=0.1282 | |
| **Seizures, n (%)** | No | 11 (91.7) | 10 (50.0) | 8 (80.0) | 9 (50.0) |
|  | Yes | 1 (8.3) | 10 (50.0) | 2 (20.0) | 9 (50.0) |
| Analysis of ‘No’ vs ‘Yes’ |  | p=0.0232 | | p=0.2264 | |
| **Walking: Short Distances, n (%)** | Normal | 10 (83.3) | 4 (20.0) | 9 (90.0) | 5 (27.8) |
|  | Minimal/moderate impairment | 2 (16.7) | 13 (65.0) | 1 (10.0) | 11 (61.1) |
|  | Severe impairment | 0 (0) | 3 (15.0) | 0 (0) | 2 (11.1) |
| Analysis of ‘Normal‘ vs ‘Issues‘ |  | p=0.0008 | | p=0.0044 | |
| **Walking: Moderate Distances, n (%)** | Normal | 9 (75.0) | 2 (10.0) | 8 (80.0) | 3 (16.7) |
|  | Minimal/moderate impairment | 3 (25.0) | 11 (55.0) | 2 (20.0) | 9 (50.0) |
|  | Severe impairment | 0 (0) | 4 (20.0) | 0 (0) | 4 (22.2) |
|  | Not able | 0 (0) | 3 (15.0) | 0 (0) | 2 (11.1) |
| Analysis of ‘Normal‘ vs ‘Issues‘ |  | p=0.0003 | | p=0.0033 | |
| **Walking: Longer Distances, n (%)** | Normal | 6 (50.0) | 1 (5.0) | 6 (60.0) | 1 (5.6) |
|  | Minimal/moderate impairment | 5 (41.7) | 9 (45.0) | 4 (40.0) | 8 (44.4) |
|  | Severe impairment | 1 (8.3) | 4 (20.0) | 0 (0) | 4 (22.2) |
|  | Not able | 0 (0) | 6 (30.0) | 0 (0) | 5 (27.8) |
| Analysis of ‘Normal‘ vs ‘Issues‘ |  | p=0.0057 | | p=0.0033 | |
| **Cognitive Delays, n (%)** | No | 8 (66.7) | 2 (10.0) | 8 (80.0) | 2 (11.1) |
|  | Yes | 4 (33.3) | 18 (90.0) | 2 (20.0) | 16 (88.9) |
| Analysis of ‘No’ vs ‘Yes’ |  | p=0.0015 | | p=0.0005 | |
| **Language Delays, n (%)** | No | 7 (58.3) | 5 (25.0) | 6 (60.0) | 4 (22.2) |
|  | Yes | 5 (41.7) | 15 (75.0) | 4 (40.0) | 14 (77.8) |
| Analysis of ‘No’ vs ‘Yes’ |  | p=0.1297 | | p=0.0974 | |

^1^NBS applies if an investigator stated diagnosis was through newborn screening or if age at diagnosis was 0.3 years or less.
^2^Early intervention defined as 0 years at start of protein restriction, Late intervention defined as ≥ 1years at start of dietary restriction
p-values from Fisher’s exact test
